# Twin pregnancy in the unicornuate uterus and non-communicating rudimentary horn: A case report

**DOI:** 10.18502/ijrm.v17i1.3822

**Published:** 2019-03-07

**Authors:** Leili Hafizi, Nayereh Ghomian

**Affiliations:** Department of Obstetrics and Gynecology, Faculty of Medicine, Mashhad University of Medical Sciences, Mashhad, Iran.

**Keywords:** *Twin pregnancy*, * Uterus*, * Mullerian ducts.*

## Abstract

**Background: **A unicornuate uterus is present in 0.1% of the general population. This müllerian anomaly carries significant obstetrical risk including abortion, preterm delivery, and rudimentary horn ruptures. **Case:** The patient is a 24-yr-old primigravida with 12-wk gestational age and a twin pregnancy in the unicornuate uterus and non-communicating rudimentary horn. One fetus in the unicornuate uterus and other in the rudimentary horn that was ruptured. In urgent laparotomy rudimentary horn and fallopian tube excised. Pregnancy in the unicornuate uterus was continued and at 38-wk gestational age, cesarean section due to premature rupture of the membrane was performed and then normal fetus was delivered. **Conclusion:** Twin pregnancy in a unicornuate uterus and rudimentary horn is a rare condition that carries a considerable risk to the mother. There is a need for increased awareness of this rare condition to prevent maternal morbidity and mortality.

## 1. Introduction

Congenital uterine malformation is seen in 1–10% of the general population, and it results from an abnormal formation, fusion, or reabsorption of the müllerian duct. A unicornuate uterus is present in 0.1% of the general population (1). In this anomaly, the underdeveloped or rudimentary horn may be present, and the uterus may be communicating and may contain an endometrium-lined cavity (2).

This müllerian anomaly carries significant obstetrical risks including first- and second-trimes term is carriage trimester miscarriage, malpresentation, fetal-growth restriction, preterm delivery, and rudimentary horn rupture (3). First- and second-trimester rupture of rudimentary horn is thought to be due to decreased muscle mass (4). The risk of rudimentary horn rupture is 50 to 90%, of which most occur in the second trimester of the pregnancy (5).

Here, we present a rare case of twin pregnancy in the unicornuate uterus and non-communicating rudimentary horn. One fetus in the unicornuate uterus and the other one in the rudimentary horn that was ruptured and pregnancy terminated with rudimentary horn excision. Pregnancy in the unicornuate uterus was continued until 38-wk gestational age.

## 2. Case Report

A 24-yr-old primigravida was presented to our hospital with abdominal pain for 24 hr. Her last menstrual period was 12 wks ago, she had vomiting and dizziness. In history, she was married for one year and her menstrual cycles were regular and with dysmenorrhea.

The patient did not have any medical, family, or psychological history including relevant genetic information. She was pale on clinical examination. Her pulse rate was 110 beats per minute and her blood pressure was 90/50 mmHg.

The abdomen had generalized tenderness and fundal height was 14 wk. There was slight vaginal bleeding and uterus was tender on vaginal examination. Posterior fornix was full and cervical motion tenderness was present. In the vaginal examination, the cervix was single and the vagina was normal.

**Figure 1:**
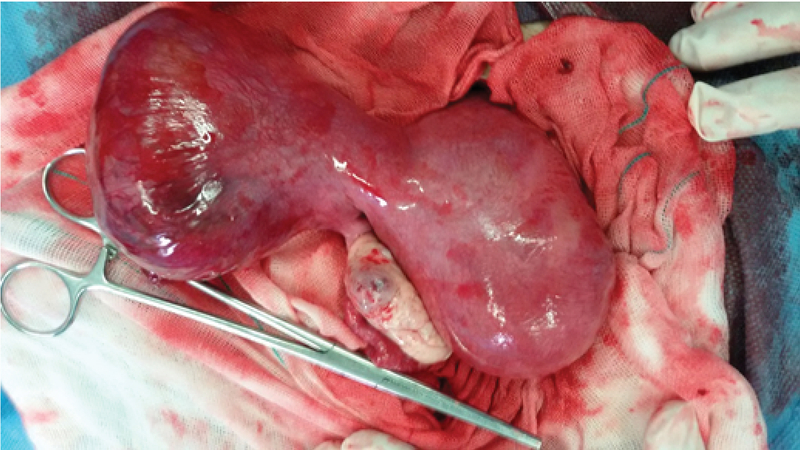
Intraoperative photograph showing the anterior view of the unicornuate uterus with the rudimentary horn.

**Figure 2:**
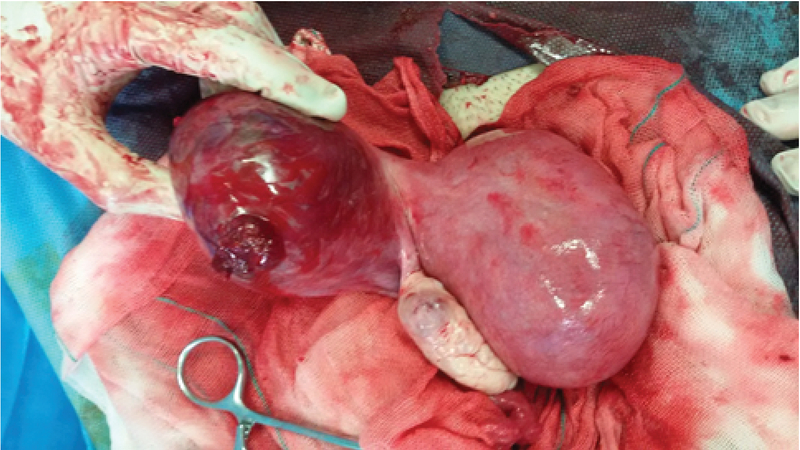
Intraoperative photograph showing the posterior view of the unicornuate uterus with the rudimentary horn having a 2-cm rupture with the placenta protruding.

Bedside ultrasonography showed hemoperitoneum and a viable 12-wk twin pregnancy: one fetus in the unicornuate uterus and other in the rudimentary horn. Urgent laparotomy was done. Intraoperatively there were 1 L of hemoperitoneum. Unicornuate uterus with the non-communicating rudimentary horn was seen. Rupture of 2cm size in left rudimentary horn with active bleeding was present on posterior margin (Figures 1, 2).

The left fallopian tube was normal. The left ovary was attached by its ligament to the rudimentary horn. Left ruptured non-communicating rudimentary horn and fallopian tube excised from small pedicle from main uterus and defect was repaired.

Her postoperative period was uneventful and she recovered well. Postoperative ultrasonography showed viable intrauterine fetus with normal cardiac activity and gestational age of 12 wk and 5 days. She was discharged on the fourth postoperative day. The patient was given a 26-wk follow-up appointment. At 38-wk gestational age, cesarean section was performed due to premature rupture of membrane and fetus with 2900gr weight and apgars 9/10 was delivered.

### Ethical consideration

Written informed consent was obtained from the patient for publication of this case report and any accompanying images. The article was submitted to the ethical committee of Mashhad University of MedicalSciences in Iran. Upon review, the ethical committee approved the above-mentioned protocol in its session held on `January 21, 2107'; Reference Number: IR.MUMS.REC.1395.522.

## 3. Discussion

A unicornuate uterus is one of the müllerian duct malformations that is present in 0.1% of the general population and carries significant obstetrical risks including rudimentary horn rupture (1). Although some case reports of pregnancy in a non-communicating rudimentary horn were reported (1–5), Oza and colleagues reported a case of unruptured non-communicating rudimentary horn pregnancy at 15-wk gestational age with intrauterine fetal death and induction failure where laparotomy was performed and rudimentary horn excised (6). Kumar and co-workers reported a case of ruptured rudimentary horn at 14 wks of pregnancy where excision of the rudimentary horn was done (7). Thakur and colleagues reported a ruptured non-communicating rudimentary horn pregnancy at 19 wk of pregnancy with a previous history of cesarean delivery (5). However, twin pregnancy in the unicornuate uterus and non-communicating rudimentary horn is a rare event. Most cases remain undiagnosed until it ruptures and presents as an emergency with hemoperitoneum (8). Nanda and colleagues reported a case of twin pregnancy in a unicornuate uterus with a non-communicating rudimentary horn in which each of fetus was in the separate horn. In this case, a cesarean section was performed and two siblings were delivered successfully (9). A literature search shows that more than 20 years ago, Nahum reported a patient with surviving twins: one fetus delivered first by cesarean section and the second one was born with natural vaginal delivery in eight days later (10). The usual outcome of rudimentary horn pregnancy is a rupture in the first or second trimester (11, 12). Initial ultrasound scan leads to early detection and can prevent maternal mortality and morbidity. In an acute abdomen with pregnancy, rupture of the rudimentary horn of the uterus is one of the differential diagnoses. In our patient, diagnosis of twin pregnancy in the unicornuate uterus with the rudimentary horn was established when she was 12 wk and presents as an acute abdomen. Intraoperative rupture of rudimentary horn and normal pregnancy in the unicornuate uterus was confirmed with good neonatal and maternal outcomes. After the termination of pregnancy, the patient had a live baby and, in her perspective, it was worth the risk of a major surgery during pregnancy.

## 4. Conclusion

Twin pregnancy in a unicornuate uterus and rudimentary horn is a rare condition that carries grave risk to the mother. There is a need for increased awareness of this rare condition to prevent maternal morbidity and mortality.

##  Conflict of Interest

The authors declare that they have no conflict of interests.
